# Alteration of the Antitumor Immune Response by Cancer-Associated Fibroblasts

**DOI:** 10.3389/fimmu.2018.00414

**Published:** 2018-03-01

**Authors:** Linda Ziani, Salem Chouaib, Jerome Thiery

**Affiliations:** ^1^INSERM, UMR 1186, Villejuif, France; ^2^Gustave Roussy Cancer Campus, Villejuif, France; ^3^Faculty of Medicine, University Paris Sud, Le Kremlin Bicêtre, France

**Keywords:** cancer, tumor microenvironment, cancer-associated fibroblasts, immune suppression, immunotherapy

## Abstract

Among cells present in the tumor microenvironment, activated fibroblasts termed cancer-associated fibroblasts (CAFs), play a critical role in the complex process of tumor-stroma interaction. CAFs, one of the prominent stromal cell populations in most types of human carcinomas, have been involved in tumor growth, angiogenesis, cancer stemness, extracellular matrix remodeling, tissue invasion, metastasis, and even chemoresistance. During the past decade, these activated tumor-associated fibroblasts have also been involved in the modulation of the anti-tumor immune response on various levels. In this review, we describe our current understanding of how CAFs accomplish this task as well as their potential therapeutic implications.

## Introduction

It is now well admitted that tumor progression and metastasis formation do not only depend on cancer cell genetic and epigenetic defects but are also controlled by the tumor microenvironment (TME) (1, 2). The TME or stroma is composed of cells from endothelial, mesenchymal, and hematopoietic origins embedded in a complex extracellular matrix (ECM), which enter into a dynamic crosstalk with tumor cells, suitable for tumor growth. Consequently, different elements such as angiogenesis, hypoxia, ECM remodeling, interstitial pressure, metabolism changes have received recent attention as key determinants of the TME modifying cancer cell behavior and disease progression, with potential clinical applications (2, 3). Moreover, the TME is also clearly involved in shaping the cellular fate of tumor-infiltrating lymphocytes and the efficacy of the anti-tumor immune response. Indeed, during tumor progression, tumor cells proliferate under adverse host conditions and use several survival strategies to block the action of key regulators/effectors of the immune response and to circumvent anti-tumor defenses (4–6). Besides the several known classical strategies used by tumor cells to escape immune surveillance (such as down regulation of antigen expression, resistance to cell-mediated lysis or expression/secretion of immunosuppressive molecules), it should be noted that tumor cell evasion from immunosurveillance is also under the control of the TME complexity (7–9). The ability of tumors to orchestrate an immunosuppressive microenvironment is dependent on several mechanisms ultimately leading to the inhibition of various immune effector cells [such as cytotoxic T cell (CTL) or natural killer (NK) cells] or to the recruitment and stimulation in the TME of immunosuppressive cells [such as regulatory T cells (Tregs), type II macrophages or myeloid-derived suppressor cells (MDSCs)]. In particular, among the stromal cells, activated fibroblasts that share similarities with fibroblasts stimulated by acute or chronic inflammatory signals, activated during a wound healing process and observed during tissue fibrosis, also known as myofibroblasts, play a critical role in the complex process of tumor cell-stroma interaction (10–13) and have emerged as important regulators of the anti-tumor immune response (14–16). Here, we will discuss the different mechanisms involved in the immuno-suppressive capabilities of activated fibroblasts in the TME, as well as their potential application for therapeutic intervention, especially in the field of cancer immunotherapy.

## Origin of Activated Fibroblasts in the TME and Role in Cancer Progression

Fibroblasts are spindle-shaped, non epithelial (cytokeratin^−^, E-cadherin^−^), non endothelial (CD31^−^) and non-immune (CD45^−^) cells of a mesenchymal lineage origin (vimentin^+^). In normal tissue, fibroblasts are usually considered as resting/quiescent cells with negligible metabolic and transcriptional activities (11), but with the ability to respond to growth factors to become activated. During this activation process, fibroblasts exhibit contractile activity, exert physical forces to modify tissue architecture, acquire proliferation and migration properties and become transcriptionally active leading to the secretion of several factors (cytokines, chemokines, etc.) and ECM components (17–19). The ability of resting fibroblasts to become activated was first observed in the context of wound healing (20) and subsequently in pathologic conditions such as acute or chronic inflammation or tissue fibrosis (a chronic wound healing response) (17, 21). This chronic tissue repair response also occurs in the context of cancer, considered as a “wound that never heals” (22). Indeed, emergence and/or accumulation of cancer cells in a given tissue represent a tissue injury, imitating a chronic wound healing response toward the tumor cells, also known as tumor fibrosis or desmoplastic reaction (23). Consequently, major players in tumor fibrotic microenvironment include activated fibroblasts, termed cancer-associated fibroblasts (CAFs), which represent one of the most abundant stromal cell types of several carcinomas including breast, prostate, pancreatic, esophageal, and colon cancers while CAFs are less abundant, but still present, in other neoplasias including ovarian, melanoma, or renal tumors (24). For example, in pancreatic cancer, 60–70% of the tumor tissue is composed of a desmoplastic stroma characterized by extensive collagen deposition and activated CAFs (25).

Several studies have clearly demonstrated that cancer cells can recruit and activate tissue resident fibroblasts in the stroma (26, 27). This phenomenon is mainly dependent on growth factors released by the cancer cells and also by infiltrating immune cells. In particular, transforming growth factor-β (TGF-β), platelet-derived growth factor (PDGF), epidermal growth factor (EGF), and fibroblast growth factor (FGF) secreted by tumor cells are key determinants of fibroblast activation and proliferation within the TME (28–31). Moreover, the secretion of interleukin (IL)-1β (IL-1β) by immune cells in early neoplasia has emerged as an initiator of nuclear factor-κB signaling in fibroblasts involved in their education and production of pro-tumorogenic and pro-inflammatory factors (32). Furthermore, emerging data suggest that the irreversible activation of CAFs might be driven by epigenetic alterations (33–36). Nevertheless, CAFs can also originate from other cell populations than resident fibroblasts through different mechanisms and depending on the tissue analyzed. Several other local sources of CAFs have been thus suggested. In breast, kidney, lung, and liver carcinomas, a portion of CAFs have been shown to potentially differentiate from epithelial cells *via* an epithelial-to-mesenchymal transition (EMT) (37, 38). A related process, termed endothelial-to-mesenchymal transition has been involved in the trans-differentiation of endothelial cells to a cell population with a CAF-like phenotype (39). Other cells linked to blood vessels, named pericytes, can trans-differentiate into CAFs in a PDGF-dependent manner (40). Moreover, in breast cancer, adipocytes were shown to differentiate in CAFs (41, 42). Finally, in liver and pancreas tumors, stellate cells, normally involved in organ regeneration, are involved in fibrosis preceding the occurrence of tumors, making them a possible source of CAFs (43, 44). Beyond these local sources, more distant one can be involved in CAFs recruitment/differentiation in the TME. In particular, mesenchymal stem cells, normally residing in the bone marrow, can be attracted in the TME to become an important source of CAFs (42, 45–48). Similarly, fibrocytes, a circulating mesenchymal cell population arising from monocytes precursors which are recruited to sites of chronic inflammation, can differentiate into CAFs after their recruitment into the TME (46, 49).

**Figure 1:**
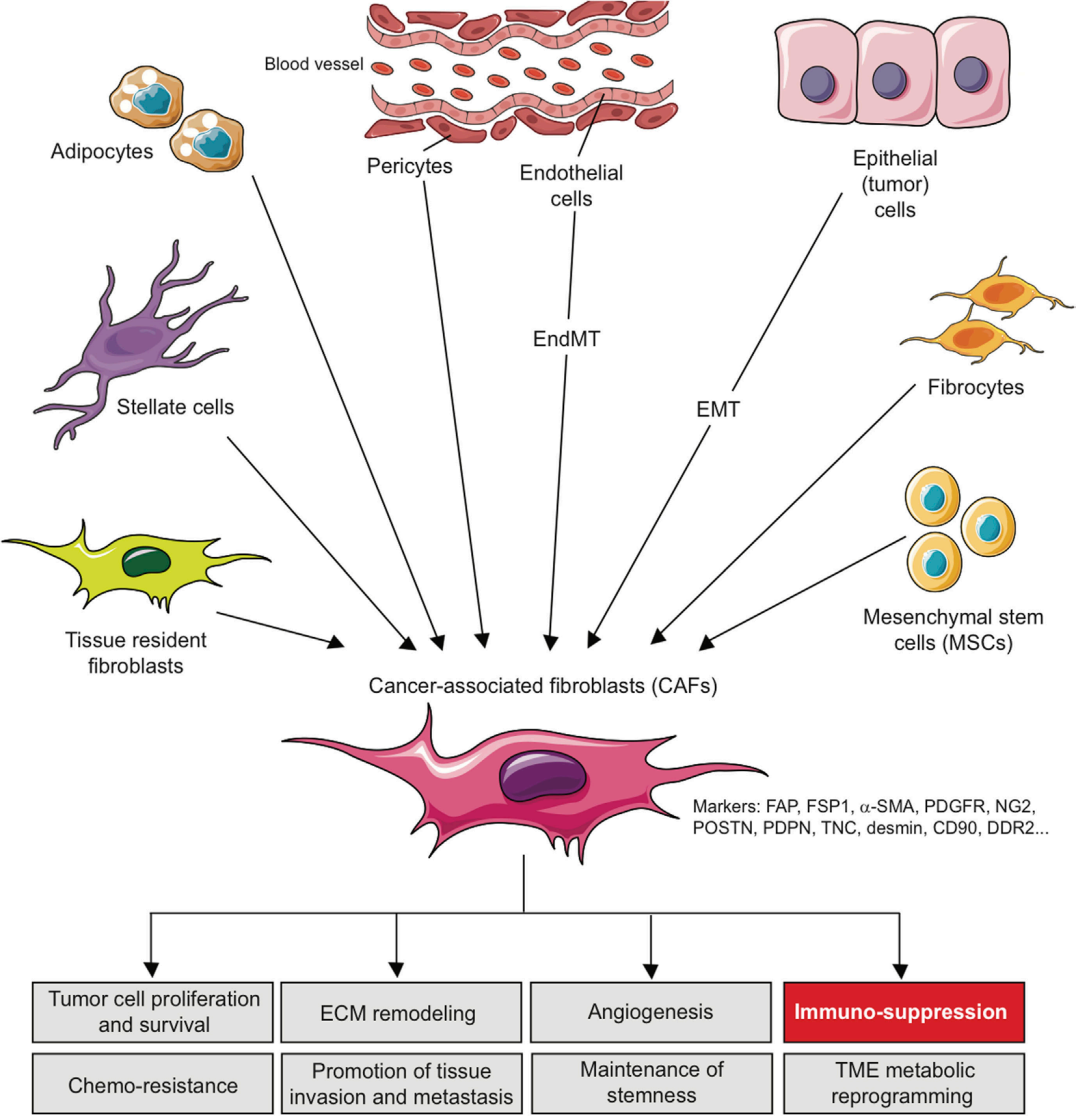
Origins of cancer-associated fibroblasts in the tumor microenvironment (TME) and role in cancer progression. CAFs can originate from diverse cell populations through different mechanisms and depending on the tissue analyzed. Local sources of CAFs include activated tissue resident fibroblasts, trans-differentiated epithelial or endothelial cells resulting from an epithelial-to-mesenchymal transition (EMT) or an endothelial-to-mesenchymal transition (EndMT), trans-differentiated pericytes, adipocytes or stellate cells. Beyond those local sources, more distant one can be involved in CAFs recruitment/differentiation in the TME, including mesenchymal stem cells, normally residing in the bone marrow, and fibrocytes. The acquisition of a CAF phenotype is associated with the potential expression of a variety of CAF-related markers as indicated. In the TME, CAFs can affect several processes leading to tumor growth, as indicated, including immuno-suppression.

These various sources represent an important determinant that contributes to the heterogeneity of CAFs (Figure 1) and makes them difficult to distinguish from other cell types present in TME. In this context, morphology and spatial distribution are key determinants in order to identify fibroblasts in a resting or activated state (11). Different markers, which are lower or not expressed by their normal counterparts, can also be used to identify activated fibroblasts such as α-smooth muscle actin (α-SMA), fibroblast-specific protein-1 (FSP-1; also called S100A4), fibroblast-activation protein (FAP), PDGF receptors (PDGFR) α or β, neuron-glial antigen-2 (NG2), periostin (POSTN), podoplanin (PDPN), tenascin-C (TNC), desmin, CD90/THY1, or discoidin domain-containing receptor 2 (DDR2) (24, 50–57). However, it is crucial to note that none of these markers is specific for normal or activated fibroblasts, and that many activated fibroblasts may not express all of these markers at the same time, most likely reflecting the high degree of heterogeneity of CAFs in the TME, as well as possible different and opposite functions in the context of specific TMEs (24). It is indeed conceivable that, depending of the context, quiescent fibroblasts or the other cell types mentioned above might be capable of differentiating into distinct subsets of functional CAFs, with possible diverse functions, either pro- or anti-tumorigenic, as observed for type I and type II macrophages (11, 58). In other words, even if a large body of literature currently supports the tumor-promoting effect of CAFs, some evidence also suggests that CAFs might also restrain tumor growth. For example, the depletion of α-SMA^+^ CAFs in pancreatic cancer accelerates tumor growth, induces immunosuppression by increasing the number of CD4^+^Foxp3^+^ Tregs in tumors and reduces survival (59). Similarly, the deletion of sonic hedgehog, a soluble ligand overexpressed by neoplastic cells in pancreatic ductal adenocarcinoma which drives the formation of a fibroblast-rich desmoplastic stroma, increases the aggressiveness of tumors (60). Nevertheless, for simplicity, we will focus the following part of this review on the tumor-promoting and immunosuppressive capabilities of CAFs, unless otherwise stated.

In the tumor stroma, CAFs interact with tumor cells and other cell types and as a sign of their activation secrete several factors such as ECM proteins (e.g., collagens), ECM-remodeling enzymes such matrix metallo-proteinases (MMPs), proteoglycans (e.g., laminin, fibronectin), chemokines [e.g., C-X-C motif chemokine ligand 2 (CXCL2), CXCL12/SDF1, chemokine ligand 2 (CCL2/MCP-1), and CCL5/Rantes], vascularization promoting factors [e.g., vascular endothelial growth factor (VEGF)] and other factors/proteins which affect tumor cells proliferation, invasiveness, survival, cancer cell metabolism, and stemness [e.g., TGF-β, EGF, FGF, hepatocyte growth factor (HGF)]. Consequently, CAFs have been involved in tumor growth, cancer cell survival, angiogenesis, maintenance of cancer stemness, ECM remodeling, tissue invasion, metastasis, metabolic reprograming of the TME and even chemoresistance [see Ref. (10–13, 24, 61) for review] (Figure 1). During the past few years, these activated tumor-associated fibroblasts have also been involved in the modulation of the anti-tumor immune response by the secretion of immunosuppressive and pro-inflammatory factors, chemokines, and chemical mediators in the TME. As such, CAFs can potentially affect both innate and adaptive antitumor immune response and consequently tumor progression.

## CAF-Mediated Regulation of the Innate Anti-Tumor Immune Response

**Figure 2:**
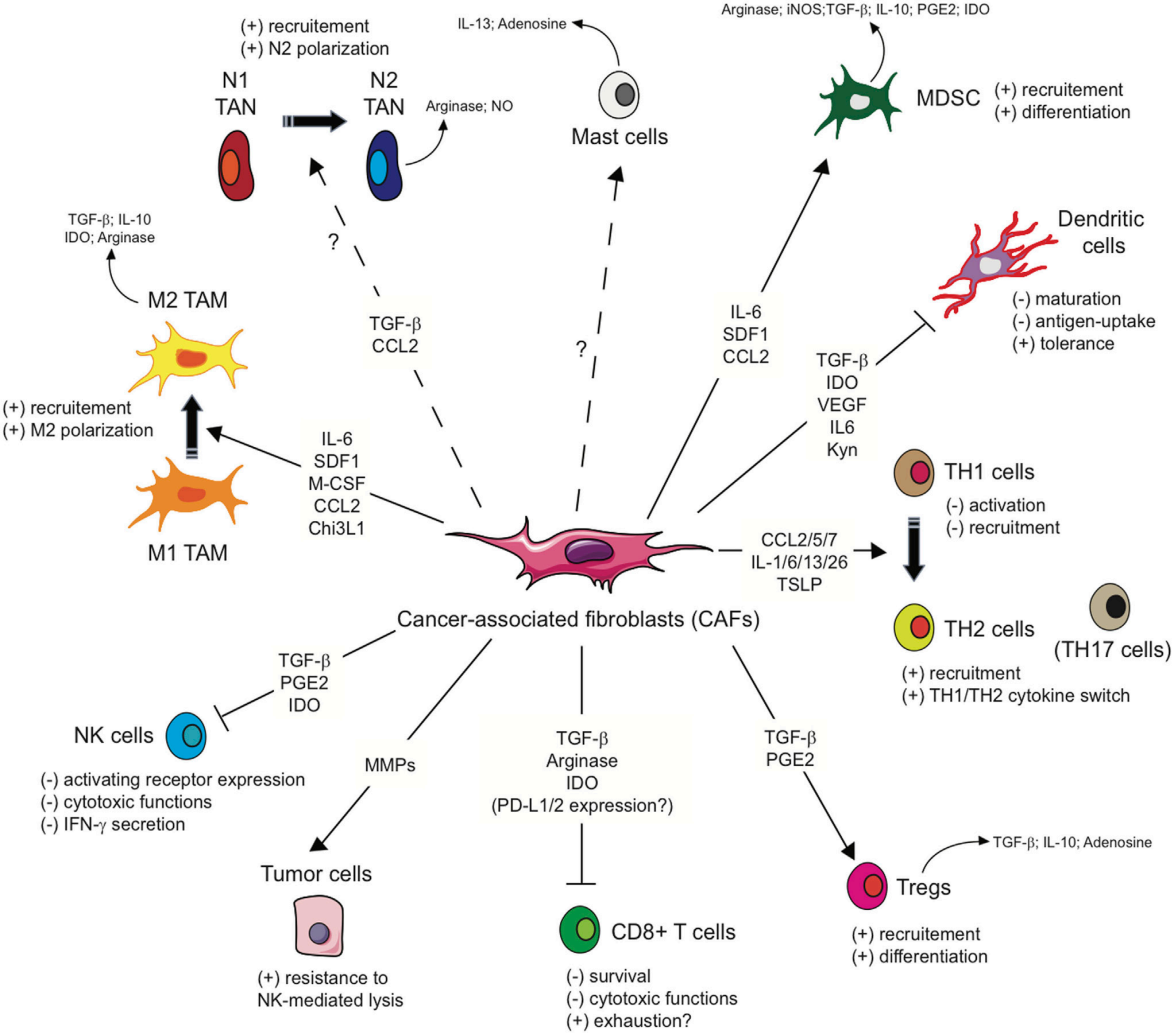
Influence of cancer-associated fibroblasts on the regulation and function of immune cells involved in the antitumor immune response. Due to their secretion of the indicated cytokines, chemokines, or other soluble factors, cancer-associated fibroblasts (CAFs) shape the tumor microenvironment and influence both the innate and adaptive anti-tumor immune response. CAFs favor the recruitment of innate immune cells, such as tumor-associated macrophages (TAM) or potentially tumor-associated neutrophils (TAN), and their acquisition of an immunosuppressive phenotype (M2 and N2, respectively), affect cytotoxic function and cytokine production of natural killer (NK) cells, as well as the susceptibility of tumor cells to NK-mediated lysis, and activate mast cells with a potential immunosuppressive phenotype. CAFs favor the recruitment and differentiation of myeloid-derived suppressor cells (MDSCs) and regulatory T cells (Tregs) and interfere with the maturation and function or dendritic cells. CAFs have also the potential ability to influence CD4^+^ Helper T (T_H_) lymphocytes, favoring tumor-promoting TH2 and TH17 responses, and reduce the activation, functions, and survival of CD8^+^ cytotoxic T cells.

As mentioned above, several studies including gene signature or mass spectrometry analysis (62–66) have shown that CAFs exhibit a particular immunomodulatory secretome including, but not limited to, CXCL1, CXCL2, CXCL5, CXCL6/GCP-2, CXCL8, CXCL9, CXCL10, CXCL12/SDF1, CCL2/MCP-1, CCL3, CCL5/Rantes, CCL7, CCL20, CCL26, IL-1β, IL-6, IL-10, VEGF, TGF-β, indoleamine-2,3-dioxygenase (IDO), prostaglandin (PG) E2 (PGE2), tumor necrosis factor (TNF) or nitric oxide (NO). This secretion profile is thought to be a major player in shaping the TME, with multiple roles in tumor progression, but beyond its role on tumor cells, this CAFs-related secretome can potentially regulate the innate immune response in several ways (Figure 2).

In particular, CAFs are important players affecting another major stromal component within tumors, known as tumor-associated macrophages (TAMs) (67). Macrophages are mainly classified into two distinct types: “classically” activated (M1 or type I) and “alternatively” activated (M2 or type II) macrophages. M1 macrophages produce high amounts of pro-inflammatory cytokines and reactive oxygen species and have the capacity to orchestrate a T_H_1 anti-tumor immune response. On the opposite, M2 macrophages play a significant role in tumor progression, promote tissue repair and angiogenesis, and are characterized by the production of immuno-suppressive factors such as IL10, Arginase, IDO and TGF-β, which inhibit cytotoxic CD8^+^ T cell-mediated immune response in the TME (67). At least in some settings, CAFs actively promote the recruitment of monocytes to the TME and their differentiation toward M2 macrophages (68). In particular, the secretion of CXCL12/SDF1, macrophage colony-stimulating factor (M-CSF also known as CSF-1), IL-6, and CCL2/MCP-1 by CAFs actively promotes the recruitment of monocytes to the TME and their differentiation into a M2 immunosuppressive phenotype (69–74). It was also recently shown that Chitinase-3-like-1 (Chi3L1; YKL-40 in humans), a secreted glycoprotein involved in several diseases including chronic inflammatory conditions, fibrotic disorders and various types of cancer, is highly expressed in CAFs isolated from mammary tumors and pulmonary metastases in mice, and in the stromal compartment of human breast carcinomas, and enhances macrophage migration in the TME and their expression of an M2-like gene signature (75). Finally, the expression of both CAF (α-SMA^+^, FSP1^+^, and FAP^+^) and M2 macrophages (CD163^+^ and DC-SIGN^+^) markers is associated with the poor clinical outcome of colorectal cancer and oral squamous cell carcinoma patients (76, 77), suggesting an association between these two cell types.

Cancer-associated fibroblasts are also potentially involved in the recruitment of neutrophils into the TME, notably through the secretion of CXCL1, CXCL2, CXCL5, CXCL6, CXCL8, and CCL2. Tumor-associated neutrophils (TANs) have been linked to a poorer prognosis for patients with renal and pancreatic cancer; gastric, hepatocellular, colorectal, head and neck carcinomas, and melanoma (78). TAN-derived factors promote tumor cell proliferation, migration, and invasion, and also induce tumor vascularization by the production of pro-angiogenic factors. Moreover, the production of Arginase 1 (Arg 1) and NO by TANs in response to CXCL8 signaling has been linked to the inhibition of T cell functions (79, 80). Nevertheless, recent studies have suggested that TANs can be polarized to an N1 anti-tumoral or N2 pro-tumoral phenotype in the TME, as observed for TAMs. N1 neutrophils are induced upon TGF-β blockade and express immuno-activating cytokines and chemokines, low levels of Arg 1, and are able to kill cancer cells. On the opposite, N2 neutrophils are characterized by expression of CXCR4, VEGF, and MMP9 and are induced following exposure to high TGF-β levels (81) and inhibit CD8^+^ T cell function by several mechanisms (82). At this point, it is thus uncertain whether CAFs can recruit TANs and drive them to an N2 phenotype in the TME, and whether this recruitment/polarization of TANs participates to the immuno-suppressive activity of CAFs.

Another cell population has also been implicated in the complex CAFs-TME interaction. Mast cells, derived from CD34^+^/CD117^+^ pluripotent hematopoietic stem cells, are tissue resident sentinel cells that, upon activation, release a wide spectrum of chemokines and cytokines (83). Interestingly, it was demonstrated in pancreatic tumors that a complex interaction between mast cells and stellate cells (often described as CAF precursors) is able to activate mast cells, which in turn enhance CAF proliferation by their secretion of IL-13 and tryptase, favoring tumor growth (84). Of note, activated mast cells could not only increase tumor progression but might also alter the anti-tumor immune response. For example the release of free adenosine (85) or IL-13 by mast cells might, respectively, inhibit T cell function and promote M2 polarization (83, 86, 87). Mast cells can also promote the generation of highly suppressive MDSCs and Tregs in the TME (88, 89). However, whether CAF-mast cell interactions are linked to the immuno-suppressive capabilities of CAFs is also not clearly established and requires further investigations.

Finally, CAFs can also affect the activity of major innate effector cells, NK cells, which participate to the early immune response through their cytotoxic activity and contribute to the adaptive immune response by the secretion of cytokines and by the promotion of antigen-presenting cell maturation. As previously mentioned, CAFs are thought to be an important source of TGF-β in the TME (90, 91). TGF-β has been involved in the decrease of NK cell activation and cytotoxic activity (92). In this regard, TGF-β-induced miR-183 inhibits DAP12 transcription (a key accessory protein for relaying signals by NK cell receptors) and reduces the expression of the NK-activating receptor NKp30 and NK Group 2D (NKG2D) (93–95), resulting in a weak NK cell cytotoxic activity in the TME. TGF-β also reduces IFN-γ secretion by NK cells, which is important for stimulating effector CD4^+^ T_H_1 cells that are required for clearing tumors, notably by repressing T-bet expression through Smad 3 (96–98). Moreover, studies involving melanoma, hepatocellular, and colorectal carcinoma-derived fibroblasts have shown that CAFs can decrease the expression of several NK activating receptors (including NKp30, NKp44, and NKG2D) on the NK cell surface, as well as perforin and granzyme B expression, through the secretion of PGE2 and/or IDO (99–101) leading to an attenuated cytotoxic activity of NK cells against their tumor target cells. We also recently demonstrated that CAFs isolated from melanoma decrease the susceptibility of melanoma tumor cells to NK cell-mediated lysis through the secretion of active MMPs which cleave two ligands of the NK-activating receptor NKG2D, MHC class I-related chain (MIC)-A and MIC-B, at the surface of the tumor cells and consequently decrease the NKG2D-dependent cytotoxic activity of NK cells against melanoma tumor cells, as well as their secretion of IFN-γ (102).

In conclusion, due to their secretion of cytokines, chemokines, or other soluble factors, CAFs shape the TME and favor the recruitment of innate immune cells, such as monocytes or neutrophils, and their acquisition of an immunosuppressive phenotype, but also affect cytotoxic function and cytokine production of NK cells.

## CAF-Mediated Regulation of the Adaptive Anti-Tumor Immune Response

Based on the immunomodulatory secretome mentioned above, CAFs might also interfere with the adaptive anti-tumor immune response at different levels, leading to a disruption of T cell function in the TME (Figure 2).

In the TME, dendritic cells (DCs), the most important antigen-presenting cell population, have a pivotal role for the activation of T cell-mediated anti-tumor immunity (103). DC biology can potentially be affected by the CAF secretome in several ways. In particular, CAF-derived TGF-β can affect DC function (96). In response to TGF-β, DCs downregulate the expression of MHC class II molecules and of the co-stimulatory molecules CD40, CD80, and CD86, which are necessary for efficient antigen presentation, and of TNF-α, IFN-γ, and IL-12, that promote T cell recruitment and survival. The resulting immature or tolerogenic DCs alter CD8^+^ cytotoxic T cell activation and the T_H_1 polarization of CD4^+^ helper T (T_H_) cell populations and also promote the formation of CD4^+^FoxP3^+^ Treg cells that potently inhibit the function of other T cells (104, 105). CAFs can also secrete IL-6 and could affect DC functions through this way. Indeed, IL-6-mediated activation of the STAT3 pathway has been involved in the alteration of the DC maturation, disabling T cell activation and inducing T cell anergy and immune tolerance (106–108). Fibroblast-produced IL-6 was also reported to favor the emergence of TAMs from monocytes at the expense of DCs (69). Expression of tryptophan 2,3-dioxygenase (TDO2) by CAFs isolated from lung cancer also promotes tryptophan degradation in kynurenines (Kyn) that inhibits DCs differentiation and functions (109). Finally, CAF-derived VEGF, in addition to its pro-angiogenic effect, has multiple immunoregulatory roles (110). In particular, VEGF inhibits DC generation and maturation (111–114), notably by reducing their MHC class II expression and their ability to take up antigens.

The role of CAFs in regulating T cell activity and function in the TME has also been suggested by several studies. As mentioned earlier, CAFs can be an important source of TGF-β in the TME, which may act on both CD8^+^ and CD4^+^ T cells (96, 105). For example, TGF-β promotes cell death of effector CD8^+^ T cells by inhibiting expression of the pro-survival protein Bcl-2 (115). TGF-β also directly alters cytotoxic CD8^+^ T cell function by inhibiting the expression of key genes involved in their cytototoxic activity, including perforin, granzymes A and B, Fas ligand, and IFN-γ (116, 117). Furthermore, CAFs could also impair T cell proliferation and effector function through other mechanisms (118), notably depending on their production of metabolic reprogramming factors. The secretion by CAFs of IDO1 (119, 120), an immuno-regulatory enzyme, might contribute to immuno-suppression, tolerance, and tumor escape by catabolizing tryptophan degradation into kynurenines (Kyn), creating an immunosuppressive TME resulting in T-cell anergy and apoptosis through depletion of tryptophan and accumulation of immunosuppressive tryptophan catabolites (121, 122). Similarly, the secretion by CAFs of Arginase 2 (Arg 2), an enzyme metabolizing l-Arginine to l-Ornithine and urea, might participate to the deprivation of Arginine in the TME, which is in normal conditions important for T cell proliferation and functions (123). In this regard, pancreatic cancer suffering patients with CAFs expressing high levels of Arg 2, especially in hypoxia-inducible factor (HIF)-1α positive hypoxic zones, demonstrate a poor clinical outcome (124). CAFs can also secrete galectins, a class of carbohydrate binding proteins that have a high affinity for β galactosides (125, 126), which possess immunoregulatory properties (127) such as, for Galectin-1, induction of apoptosis of activated T cells by binding the glycoprotein receptors CD7, CD43, and CD45 on the cell surface (128, 129). Finally, the secretion of CXCL12/SDF-1 by CAFs from lung and pancreatic tumors can contribute to the exclusion of T cells from the cancer cell proximity (130).

Cancer-associated fibroblasts have also the potential ability to influence CD4^+^ Helper T (T_H_) lymphocytes, switching them from anti-tumor to pro-tumor cells. CD4^+^ T_H_ cells can differentiate into multiple sublineages with different functions and cytokine secretion profiles, which in turn can induce, maintain or regulate antitumor immune responses (131). Schematically, naïve CD4^+^ T cells can differentiate into T_H1_ cells mainly secreting IFN-γ and promoting CD8^+^ T cell-dependent immune response, or into T_H2_ cells mainly secreting IL-4 and orchestrating humoral immunity. In terms of antitumor immune responses, the superior effects of T_H_1 cells are thought to be the result of the production of large amounts of IFN-γ, as well as chemokines, which enhance the priming and expansion of antitumor CD8^+^ cells and help to recruit NK cells and type I macrophages to tumor sites. A third major effector population of CD4^+^ T cells that could be derived from naïve CD4^+^ T cells was also shown to exist. These cells, designated T_H_17 cells (132, 133), are characterized by the production of IL-17 and IL-22 and might have, at least under some circumstances, pro-tumor and immunosuppressive functions in the TME (134), even if this particular point remains highly controversial. Finally, under tolerogenic conditions, naïve CD4^+^ T cell precursors can differentiate into inducible Tregs that upregulate the expression of the FoxP3 transcription factor (135). Depending on the tumor type, Tregs can be highly enriched in the TME, limiting antitumor immune responses and promoting immunological ignorance of cancer cells, especially through the secretion of immunosuppressive cytokines (TGF-β, IL-10…) (136). In the TME, the presence of CAFs and their secretion of CCL2, CCL5, and CCL17 as well as the polarizing cytokines IL-1, IL-6, IL-13, and IL-26 can favor a tumor promoting T_H_2 and T_H_17 immune response, as the expense of tumor protective T_H_1 response (32, 137–139). For example, in a murine model of breast tumor, the elimination of CAFs *in vivo* by a DNA vaccine targeting FAP resulted in a shift of the immune TME from a T_H_2 to a T_H_1 polarization. This shift was characterized by an increased expression of IL-2 and IL-7, an increased of CD8^+^ T cell population, and a diminished recruitment of TAM, MDSC, and Tregs (139). Moreover, in pancreatic cancer, the secretion of thymic stromal lymphopoietin (TSLP) by CAFs has been associated with a T_H_2 cell polarization through myeloid DC conditioning (140). As a main source of TGF-β in the TME, CAFs can also promote Tregs recruitment and differentiation (141). Of note, it has been suggested that CAFs and Tregs enter to a cross-talk *via* their reciprocal expression of TGF-β, increasing both CAFs activation and Tregs activity. In this regard, FoxP3^+^ Tregs coexisting with CAFs are correlated with a poor outcome in lung adenocarcinoma (142). Moreover, it was shown that the expression of cyclo-oxygenase-2 (COX-2) by CAFs in lung or pancreatic cancers leads to their secretion of PGE-2, which plays an essential role in Tregs functionality by inducing FoxP3 expression (143, 144).

Cancer-associated fibroblasts in the TME can also interfere with the T cell-dependent immune response by modulating MDSCs. MDSCs are a heterogeneous population of immature myeloid cells that accumulate during pathologic conditions, such as cancer (145, 146). The main factors involved in MDSC-mediated immune suppression include the secretion of Arginase, iNOS, TGF-β, IL-10, PGE2 and IDO, regulating DC and T cell functions, as well as NK cells and macrophages. It has been demonstrated that CAFs isolated from pancreatic tumors drive monocyte precursors toward an MDSC phenotype, in a STAT3-dependent manner, through their secretion of IL-6 (72, 147). Similarly, CAFs from hepatic carcinomas attract monocytes to the TME by their secretion of CXCL12/SDF1 and induce their differentiation into MDSCs through IL-6-mediated STAT3 activation (148), thus altering T cell proliferation and functions, as well as the patients overall survival. Pancreatic stellate cells (described as CAFs precursors) also produce MDSC-promoting cytokines (IL-6, VEGF, M-CSF) and chemokines (CXCL12/SDF1, CCL2/MCP-1) and similarly promote differentiation of MDSCs in a STAT3-dependent manner (72). In a murine liver tumor model, it was also shown that FAP^+^ CAFs are a major source of CCL2 and that fibroblastic STAT3-CCL2 signaling promotes tumor growth by enhancing the recruitment of MDSCs, which also predicts poor prognosis of patients with intrahepatic cholangiocarcinoma (149).

Finally, an interesting but still controversial point was recently raised based on the observation that CAFs from colon and lung cancers or from melanoma might express programmed death-ligand-1 (PD-L1) and/or PD-L2 (150–152). PD-L1 and PD-L2 are members of the B7 family of co-stimulatory/co-inhibitory molecules expressed by a wide range of cancer cells and engage their receptor programmed death receptor 1 (PD1) expressed on T-cells, strongly counteracting TCR signaling and CD28-co-stimulation (153), resulting in the inhibition of T cell activation, proliferation, and functions. As such, therapeutic antibodies that block PD-L1/PD1 interactions between cancer cells and T cells have recently received great attention because of their capacity to reverse T cell exhaustion in response to persistent antigen stimulation and to improve the immune control of cancer in a variety of tumor types, including melanoma, lung, and renal cell carcinomas (154). As mentioned above, it was shown that myofibroblasts/CAFs from colon cancer expressed PD-L1 and PD-L2 and negatively regulate CD4^+^ T_H_ cell proliferative response (152). Similarly, CAFs isolated from lung carcinoma were shown to constitutively express PD-L1 and PD-L2, which can be upregulated by IFN-γ, and negatively regulate tumor-associated CD8^+^ T cell activation (151). In melanoma, PD-L1 expression on CAFs seems to be dependent of IL-1α/β secreted by melanoma tumor cells and melanocytes and could participate to the suppression of melanoma-specific CD8^+^ T cells (150). However, most of these discoveries rely on CAFs isolation and *in vitro* experiments, with potential artifacts (155), and clearly require further investigations to determine the physiological relevance of potential PD-L1/L2 expression by CAFs on their immunosuppressive capabilities *in vivo*.

In conclusion, the CAF secretome can shape the T cell-dependent antitumor immune response by affecting several populations such as DCs, MDSCs, by switching CD4^+^ T_H_ lymphocytes from a T_H1_ to a T_H2_ phenotype, by affecting Tregs and T_H_17 cells, by affecting CD8^+^ T cell functions or eventually by expressing some ligands of immune checkpoint receptors.

## Indirect Effect of CAFs on Anti-Tumor Immune Response

As mentioned earlier, CAF activation in the TME results in a remodeling of the ECM through deposition of several components and by proteolytic degradation, which in turn affect tumor behavior (18, 156, 157). For example, increased ECM rigidity resulting from thickening and linearization of collagen fibers has been shown to regulate tumor growth and metastasis (158, 159). This modified ECM protein network is also presumed to restrict access of immune cells to cancer cells, serving as a physical barrier at least in some models (160, 161). As such, CAF-modified ECM might be involved in T cell exclusion from the proximity of cancer cells, which has been shown as a dominant immunosuppressive mechanism in multiple cancers and a predictor of patient clinical outcome (160). In this regard, in pancreatic tumor models, it has been proposed that when fibrosis is extensive, the “scar-like” ECM may act as a barrier for CTL infiltration into tumors (162). It was also found that focal adhesion kinase [FAK; a crucial signaling protein that is activated by numerous stimuli and functions as a biosensor to control cell motility (163)] activity is elevated in human pancreatic ductal adenocarcinoma tissues and correlates with high levels of fibrosis and poor CD8^+^ CTL infiltration (164). Similarly, in lung cancers, CAFs could restrict CD4^+^ and CD8^+^ T cells motility. Indeed, it was observed an active T cell motility in loose fibronectin and collagen regions, whereas T cells poorly migrate in dense matrix areas. Furthermore, aligned fibers in perivascular regions and around tumor epithelial cell regions dictate the migratory trajectory of T cells and restricted them from entering tumor islets (165, 166). Finally, interactions between tumor cells and the surrounding modified ECM have been involved as primary forces driving the EMT process. Consequently, the imbalanced biomechanical force at the tumor-stroma interface is an important player initiating EMT (167), which can subsequently lead to tumor cells escaping from T cell-mediated lysis after their acquisition of a mesenchymal-like phenotype (168–170). Thus, in the region where the ECM has been extensively modified by CAFs, an EMT process could protect tumor cells from T cell-mediated destruction.

The CAF-mediated remodeling of the ECM might also affect other immune population than T cells. For example, CAFs have been identified as an important source of hyaluronan, also called hyaluronic acid, a component of the ECM which promotes TAM recruitment, as the genetic ablation of the hyaluronan synthase strongly diminishes their presence within the TME (171). In pancreatic and breast cancers, it was also found that extensive deposition of type I collagen, which can be highly secreted by CAFs, improves TAM infiltration (172), with a potential effect of the ECM composition on their M2 polarization (173, 174). The high levels of CAF-secreted collagen I in tumors could also activate leukocyte-associated Ig-like receptor (LAIR)-1, a collagen-receptor that inhibits immune cell function upon collagen binding (175). Nevertheless, the regulation of macrophages polarization by the ECM composition, as well as its effect on, but not limited to, MDSC, neutrophils, or DCs is still poorly understood.

In addition to the extensive remodeling of the ECM, CAFs might also indirectly regulate the anti-tumor immune response by participating in the emergence of hypoxic stress within the TME. Indeed, in tumors with a high level of fibrosis, tumor tissues are often poorly oxygenated, with a limited number of functional blood vessels, resulting in the presence of zones with a low oxygen pressure called “hypoxic zones” (16, 176, 177). Even if, as mentioned above, CAFs are described as regulators of angiogenesis through the secretion of pro-angiogenic factors, such as VEGF or through the recruitment of endothelial progenitors in the tumor through the release of SDF-1 in the TME (178), the blood vessels present in the TME are poorly functional and leaky. The resulting leaky vessels not only trigger a high interstitial fluid pressure in the TME which affect immune cell transmigration from the vessels to the TME (179), but also affect oxygen availability and acidification of the TME (180). In other words, by their global action on the TME, the presence of CAFs might participate to abnormal angiogenesis and to the creation of hypoxic zones that contribute to the immunosuppressive network within the TME. Indeed, hypoxia has been found to impair the antitumor immune response by several mechanisms (181–184), such as alteration of NK and T cell activation and effector functions, induction of PD-L1 expression on MDSCs *via* HIF-1α transcription factor, and attraction of TAMs or Tregs to the tumor bed. Furthermore, hypoxic tumor cells secrete factors including TGF-β and PDGF that promote conversion of precursor cell types into CAFs (185), and it was also shown that stromal fibroblasts synergize with hypoxic stress to enhance melanoma aggressiveness (186). This indicates a potential role of hypoxia in the CAFs activation, either by directly acting on CAFs or indirectly by acting on tumor cells, or in their function in the TME. Thus, one may consider that hypoxia not only promotes CAFs activation but might also increase their immunosuppressive properties, even if this last particular point needs to be clarified.

Overall, CAFs might indirectly affect the anti-tumor immune response, with many described and not yet elucidated distinct possibilities, such as the modification of the ECM, vasculature or architecture of the tumors, which make this field very challenging.

## Targeting CAFs to Improve Anti-Tumor Immune Response and Immunotherapy

Given the fact that CAFs impair the anti-tumor immunity (and more generally exert pro-tumorigenic effects) by several mechanisms, the design of pre-clinical or clinical studies in order to target these cells in the TME is very seductive to amplify the antitumor immune response and to develop “anti-CAF”-based immunotherapeutic approaches. Such studies can be envisioned based on agents directly targeting CAF specific proteins (e.g., FAP…) and signaling pathways involved in CAF activation (e.g., TGF-β, PDGF, FGF…) or less specifically targeting CAF-secreted factors. Potential therapies aiming at targeting CAFs or reversing the CAF “state,” as well as the ongoing clinical trials have been extensively reviewed in Ref. (18).

Recently, anti-CAF therapies have been mainly focused on FAP (187). A pioneer study has shown, in a transgenic mouse model in which FAP-expressing cells can be ablated, that the depletion of FAP-expressing cells cause rapid hypoxic necrosis of both Lewis lung carcinoma and stromal cells in immunogenic tumors by a process involving IFN-γ and TNF-α, which have previously been shown to be involved in CD8^+^ T cell-dependent killing of tumor cells (188). The development of chimeric antigen receptor (CAR) T cells targeting FAP has also shown promising results in murine models (189–191) and in malignant pleural mesothelioma patient derived xenograft models (192). A recent study has also demonstrated in two murine melanoma models that depleting FAP^+^ stromal cells from the TME upon vaccination with an adenoviral-vector reduced the frequencies and functions of immunosuppressive cells, resulting in prolonged survival of melanoma-bearing mice associated with a robust CD8^+^ T cell response (193). Similarly, in LL2 (murine lung cancer), CT26 (murine colon cancer), and B16F10 (murine melanoma) models, a whole-tumor cell vaccine modified to express FAP seems to induce antitumor immunity against both tumor cells and CAFs and enhances the infiltration of CD8^+^ T lymphocytes and decreases the accumulation of immunosuppressive cells in the TME (194). Nevertheless, it should be noted that, in addition to CAFs, FAP can be expressed by cells present in several tissues, including multipotent bone marrow stem cells or skeletal muscles. As such, another study has shown that adoptive transfer of FAP-reactive CAR-T cells into mice bearing a variety of subcutaneous tumors mediated limited antitumor effects and induced significant cachexia (a syndrome of progressive weight loss, anorexia, and persistent erosion of body muscle mass) and lethal bone toxicities in two murine strains (195). Thus, these lethal bone toxicity and cachexia observed after CAR T cell-based immunotherapy targeting FAP highlight cautions against its use as a universal target.

As such, targeting the CAF “secretome” or activation pathways, in order to revert the CAF “state,” might be a safer alternative to abrogate, at least partly and probably less specifically, their immunosuppressive role in the TME. In this regard, a recent publication demonstrated that targeting CXCL12 from FAP-expressing CAFs with AMD3100 (Plerixafor) synergizes with anti-PD-L1 immunotherapy in pancreatic cancer (130). Similarly, other proteins secreted by CAFs could be also targeted in order to restrain the immunosuppressive capabilities of these cells, such as IL-6 or TGF-β, using multiple inhibitors (18). For example, trihydroxyphenolic compounds were identified as potent blockers of TGF-β1 in the presence of active lysyl oxidase-like 2 (LOXL2; a member of mammalian copper-dependent LOX enzymes only expressed by fibroblasts or cancer cells and involved in intra- and intermolecular covalent collagen cross-links), and induce potent blockade of pathological collagen accumulation *in vivo* (196). Thus, these compounds might interfere with the T cell exclusion mediated by the CAF-dependent ECM remodeling previously mentioned, even if this particular point is still hypothetical. The use of Tranilast (Rizaben) (a known suppressor of fibroblast proliferation and TGF-β secretion) has also demonstrated a synergistic effect with a DC-based vaccine in C57BL/6 mice bearing syngeneic E-G7 lymphoma, LLC1 Lewis lung cancer or B16F1 melanoma (197). Another example is retinoic acid, a small molecular derivative of vitamin A, which inhibits IL-6 and ECM production by CAFs (198), potentially affecting their immunosuppressive properties. Nevertheless, more studies are clearly needed to identify other potential therapeutic agents targeting CAFs and/or their immunosuppressive network, which might be use in combination with the current or future anti-tumor immunotherapeutic approaches.

## Concluding Remarks

Despite their relative abundance in tumors, fibroblasts have been ignored over decades, but their crucial role has now emerged in the fields of tumor biology and oncology. CAFs have pleiotropic functions in tumor growth and participate to the inflammatory phenotype of the TME by releasing a variety of chemokines, cytokines, and other factors leading to the alteration of the antitumor immune response. Nevertheless, this complex immunosuppressive network related to the “secretome” of CAFs is still poorly understood, even if extensive efforts allowed apprehending their role in both the innate and the adaptive immune response. Of note, the notion that the CAF-specific secretome modulates the anti-tumor immune response often relies on studies limited to cells expanded *in vitro*. Future challenging studies using preclinical models will be thus needed in order to define more precisely the functional list of CAF-derived factors that exert an immunomodulatory role in the context of the TME complexity *in vivo*. This is crucial in order to fully understand the global regulation of the antitumor immune response and might also lead to the identification of novel potential therapeutic targets with the ability to increase the efficiency of anti-tumor immunotherapeutic approaches. In particular, targeting the CAFs or their secretome may probably not induce a complete tumor cell death by itself, but it will help to reduce immune effector cell dysfunctions as well as the recruitment of immunosuppressive cells, thus releasing the “brake” for a more effective immune response in combination with therapy targeting immune checkpoints (e.g., anti-CTLA4, anti-PD1/PD-L1 antibodies) or other mechanisms impairing the anti-tumor immune response in patients (199).

## Author Contributions

JT wrote the manuscript. LZ and SC participate to helpful discussion and edited the manuscript.

## Conflict of Interest Statement

The authors declare that the research was conducted in the absence of any commercial or financial relationships that could be construed as a potential conflict of interest.
